# EF-Tu From Non-typeable *Haemophilus influenzae* Is an Immunogenic Surface-Exposed Protein Targeted by Bactericidal Antibodies

**DOI:** 10.3389/fimmu.2018.02910

**Published:** 2018-12-18

**Authors:** Oskar Thofte, Yu-Ching Su, Marta Brant, Nils Littorin, Benjamin Luke Duell, Vera Alvarado, Farshid Jalalvand, Kristian Riesbeck

**Affiliations:** Clinical Microbiology, Department of Translational Medicine, Faculty of Medicine, Lund University, Malmö, Sweden

**Keywords:** antibody response, elongation factor Tu (EF-Tu), epitope mapping, *Haemophilus influenzae* (Hi), immunization, rabbit, opsonophagocytosis, serum resistance

## Abstract

Non-typeable *Haemophilus influenzae* (NTHi), a commensal organism in pre-school children, is an opportunistic pathogen causing respiratory tract infections including acute otitis media. Adults suffering from chronic obstructive pulmonary disease (COPD) are persistently colonized by NTHi. Previous research has suggested that, in some bacterial species, the intracellular elongation factor thermo-unstable (EF-Tu) can moonlight as a surface protein upon host encounter. The aim of this study was to determine whether EF-Tu localizes to the surface of *H. influenzae*, and if such surface-associated EF-Tu is a target for bactericidal antibodies. Using flow cytometry, transmission immunoelectron microscopy, and epitope mapping, we demonstrated that EF-Tu is exposed at the surface of NTHi, and identified immunodominant epitopes of this protein. Rabbits immunized with whole-cell NTHi produced significantly more immunoglobulin G (IgG) directed against EF-Tu than against the NTHi outer membrane proteins D and F as revealed by enzyme-linked immunosorbent assays. Chemical cleavage of NTHi EF-Tu by cyanogen bromide (CNBr) followed by immunoblotting showed that the immunodominant epitopes were located within the central and C-terminal regions of the protein. Peptide epitope mapping by dot blot analysis further revealed four different immunodominant peptide sequences; EF-Tu^41−65^, EF-Tu^161−185^, EF-Tu^221−245^, and EF-Tu^281−305^. These epitopes were confirmed to be surface-exposed and accessible by peptide-specific antibodies in flow cytometry. We also analyzed whether antibodies raised against NTHi EF-Tu cross-react with other respiratory tract pathogens. Anti-EF-Tu IgG significantly detected EF-Tu on unencapsulated bacteria, including the Gram-negative *H. parainfluenzae, H. haemolyticus, Moraxella catarrhalis* and various Gram-positive *Streptococci* of the oral microbiome. In contrast, considerably less EF-Tu was observed at the surface of encapsulated bacteria including *H. influenzae* serotype b (Hib) and *Streptococcus pneumoniae* (e.g., serotype 3 and 4). Removal of the capsule, as exemplified by Hib RM804, resulted in increased EF-Tu surface density. Finally, anti-NTHi EF-Tu IgG promoted complement-dependent bacterial killing of NTHi and other unencapsulated Gram-negative bacteria as well as opsonophagocytosis of Gram-positive bacteria. In conclusion, our data demonstrate that NTHi EF-Tu is surface-exposed and recognized by antibodies mediating host innate immunity against NTHi in addition to other unencapsulated respiratory tract bacteria.

## Introduction

The Gram-negative bacterium *Haemophilus influenzae* is subdivided into two categories based on the presence of a polysaccharide capsule; the encapsulated *H. influenzae* is classified as serotypes a-f and unencapsulated non-typeable *H. influenzae* (NTHi). Introduction of a vaccine against *H. influenzae* type b (Hib) in the 1990s substantially reduced Hib infections. NTHi is currently the most common cause of *Haemophilus* infections in humans, and any vaccine against NTHi does not exist. The bacterium is rarely invasive, causing sepsis predominantly in the elderly or in patients with co-morbidities (1). However, NTHi is commonly associated with respiratory tract infections. Pre-school children, harboring NTHi, *Moraxella catarrhalis*, and *Streptococcus pneumoniae* as commensals, are at the highest risk. In this age group, NTHi often causes acute otitis media (AOM) and sinusitis, occasionally upon co-infection with the common cold viruses (2). In the adult population, NTHi mainly infects and persistently colonizes patients with chronic obstructive pulmonary disease (COPD) (3). However, more virulent or antimicrobial-resistant sequence types of NTHi, such as sequence type (ST) 14, can cause severe sinusitis, bronchitis, and pneumonia in healthy adults (4).

Recent research, exploring prevention of NTHi infections, has identified several protein-based NTHi outer membrane proteins that potentially also can be used as vaccine candidates (5–7). One example is the adhesin *H. influenzae* protein F that interacts with the extracellular matrix proteins laminin and vitronectin, the latter of which inhibits the terminal pathway of complement activation (8, 9). Another example is Protein D, an enzyme with glycerophosphodiesterase activity that is currently included as a carrier protein in a 10-valent conjugated pneumococcal vaccine (Synflorix®) (10, 11).

Elongation factor thermo unstable (EF-Tu) is an essential bacterial protein that constitutes up to 5% of the total cell content (12). In *E. coli*, the genes *tufA* and *tufB* encode 40- to 45-kDa EF-Tu proteins, each containing three structural domains and varying only in their C-termini (13). EF-Tu, which binds various guanosine-containing polyphosphates, functions in polypeptide elongation with aminoacyl transfer RNAs and guanosine triphosphate. Early studies have shown that EF-Tu is located at the surface in *E. coli* (14). Subsequent studies have demonstrated that EF-Tu is surface-exposed in other bacterial species, including Gram-negative *Acinetobacter baumanii, Borrelia burgdorferi* and *Pseudomonas aeruginosa* (15–17), and Gram-positive *Staphylococcus aureus* and *Streptococcus pneumoniae* (18, 19). Extracellular localization of the translation elongation factor 1 (Tef1) of *Candida albicans*, an ortholog of EF-Tu, has also been reported (20).

Extracellular EF-Tu was initially considered a contaminant from the cytoplasm due to its high abundance in the cell. However, EF-Tu was eventually recognized as a moonlighting protein playing several roles depending on the bacterial species in question. In addition to EF-Tu, other proteins initially identified as intracellular have been described to have extracellular functions (21). EF-Tu-dependent interactions with several host molecules have been verified both biochemically and functionally. For example, *P. aeruginosa*, commonly infecting chronic wounds and patients suffering from cystic fibrosis, uses EF-Tu to attract human plasma proteins such as Factor H, Factor H-like protein, and plasminogen, thereby manipulating the activation of the alternative complement pathway (17). Moreover, *S. pneumoniae* EF-Tu has been found to increase bacterial survival in the presence of host components (19). Extracellular matrix proteins represent other putative targets for bacterial EF-Tu; *Lactobacillus casei* and *Mycoplasma pneumoniae* use EF-Tu as a receptor for fibronectin (22–24).

The moonlighting function of EF-Tu in exploiting the endogenous inhibitors of the complement system represents one of the strategies used by pathogens to evade host innate immunity (17, 19). Evasion of the complement system is also important for the pathogenicity of NTHi (25). However, the host, unable to modify the innate defense system *per se*, also relies on the adaptive immune system and the generation of high-affinity antibodies. Production of a wide repertoire of antibodies against bacterial proteins, such as EF-Tu, begins in the first year after birth and continues throughout life as a strategy to defend against intruding bacteria. Interestingly, an immunoproteome analysis revealed that infection with Shiga toxin-producing *E. coli* (STEC) significantly increased levels of serum immunoglobulin G (IgG) directed against EF-Tu (26). Sera from patients suffering from meningococcal disease also contain higher concentrations of IgG against EF-Tu (27).

Considering these findings, the present study sought to determine whether EF-Tu is also present on the surface of the respiratory pathogen NTHi. Moreover, we wanted to assess whether an immune response against EF-Tu is elicited after exposure to NTHi cells. We also determined whether anti-NTHi EF-Tu IgG recognizes other bacterial species in the respiratory tract microbiome.

## Results

### Unencapsulated *Haemophilus influenzae* Displays EF-Tu at the Cell Surface

**Figure 1:**
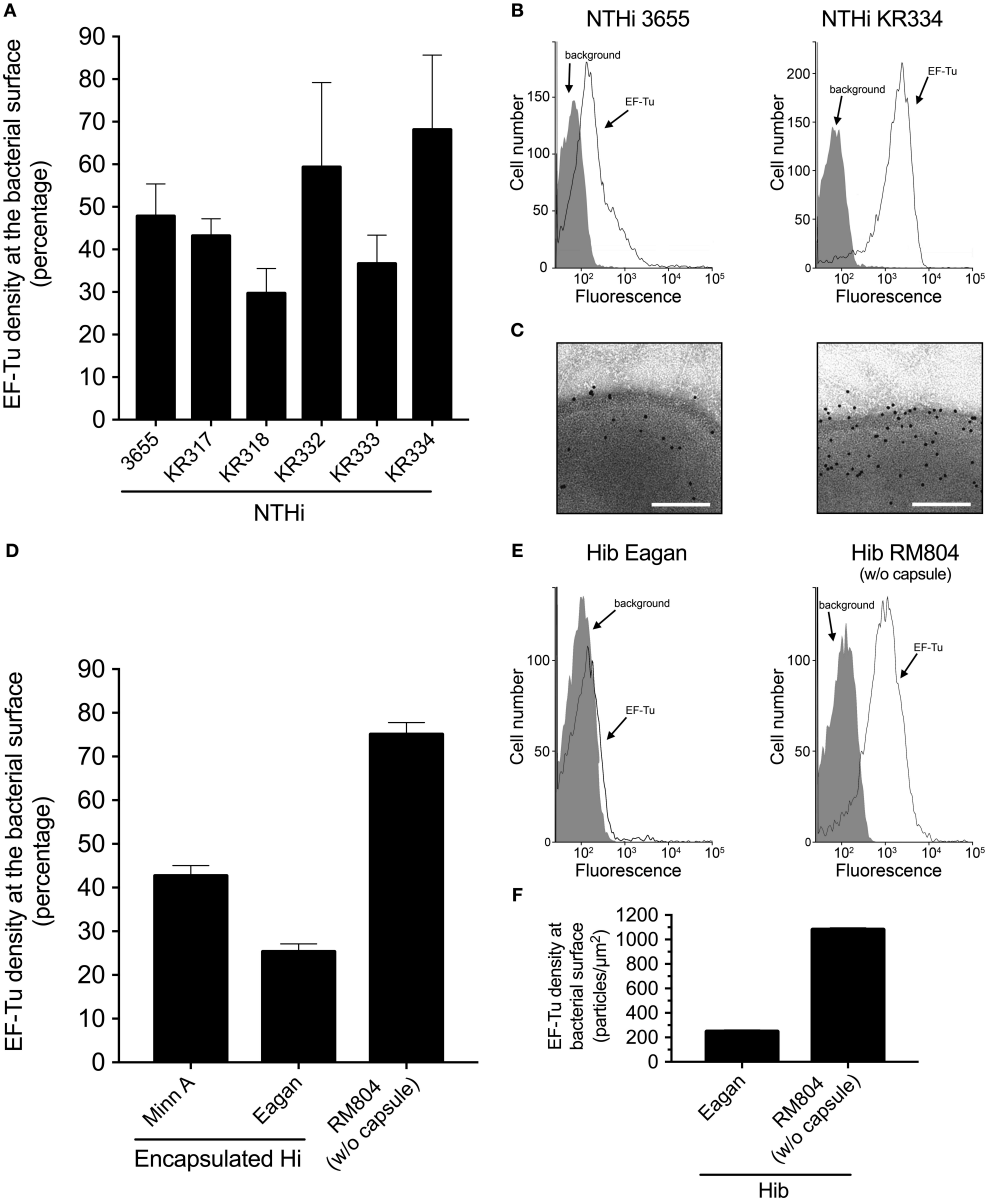
EF-Tu is present at the NTHi cell surface. **(A)** Rabbit antibodies raised against recombinant NTHi EF-Tu recognize different NTHi clinical isolates. NTHi (*n* = 6) were analyzed by flow cytometry. Mean values from three independent experiments are shown and error bars represent standard error of the mean (SEM). **(B)** Representative flow cytometry profiles of NTHi 3655 and KR334. **(C)** Localization of EF-Tu in NTHi 3655 and KR334 was analyzed by transmission electron microscopy (TEM). **(D)** Encapsulated *H. influenzae* represented by *H. influenzae* type b (MinnA and Eagan) as compared to the non-encapsulated mutant *H. influenzae* RM804 based upon Hib Eagan. **(E)** Representative flow cytometry profiles of Hib Eagan and the capsule mutant Hib RM804. **(F)** Surface concentration of EF-Tu on Hib Eagan and RM804 as measured by TEM. Anti-EF-Tu IgG was used in all experiments. Antibodies were affinity purified from rabbits that had been immunized with recombinant NTHi EF-Tu produced in *E. coli*. Flow cytometry analyses were performed using *H. influenzae* incubated with rabbit anti-EF-Tu IgG followed by secondary FITC-conjugated goat anti-IgG pAbs. Background represents bacteria incubated with only the secondary antibody, and was defined as < 2% of positive bacterial cells. Error bars indicate SEM. For TEM visualization, a gold-labeled secondary antibody was used.

To analyze whether *H. influenzae* carries EF-Tu at its cell surface, we raised anti-EF-Tu polyclonal antibodies (pAbs) by immunizing rabbits with manufactured recombinant EF-Tu derived from *H. influenzae*. Rabbit pAbs, produced as a result of an immune response elicited by recombinant EF-Tu, readily detected EF-Tu on the cell surface of clinical NTHi strains, albeit at different levels, as revealed by flow cytometry (Figures 1A,B) and transmission immunoelectron microscopy (TEM) (Figure 1C). In contrast to NTHi, encapsulated *H. influenzae* type b (Hib) strain Eagan and harbored less surface-exposed EF-Tu (Figure 1D). Hib MinnA carried, however, EF-Tu to the same level as NTHi. Importantly, removal of the capsule from Hib Eagan promoted exposure of EF-Tu, as evidenced by the unencapsulated mutant Hib Eagan designated RM804 (Figures 1E,F). These results suggested that mainly unencapsulated *H. influenzae*, that is, NTHi contains antibody-accessible EF-Tu on its outer membrane.

### EF-Tu Is Highly Immunogenic in Rabbits Immunized With Whole NTHi Cells

**Figure 2:**
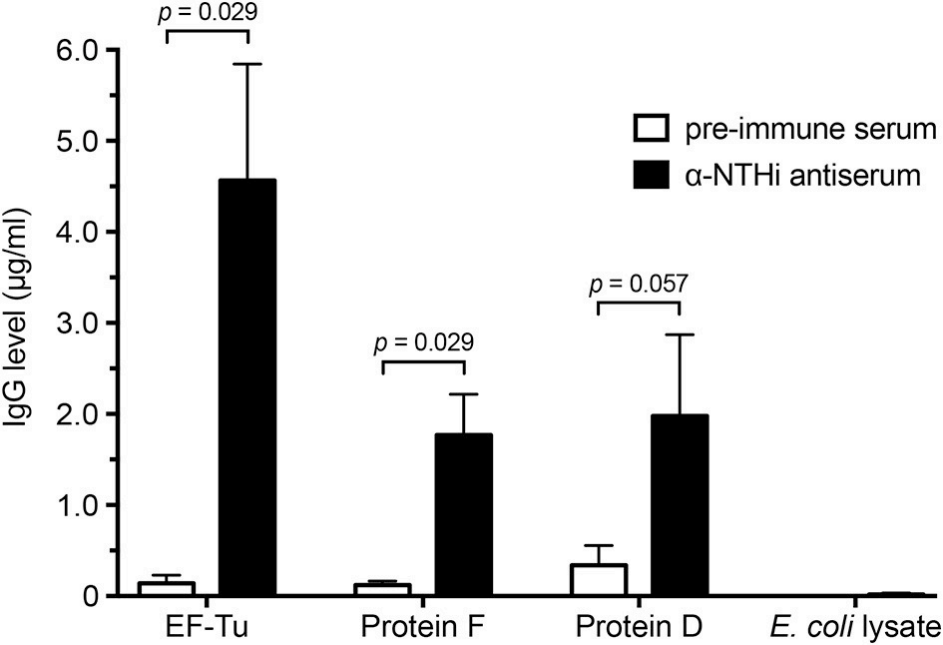
High concentrations of anti-EF-Tu IgG is produced in rabbits immunized with whole NTHi. Mean values of anti-EF-Tu IgG levels are shown for 4 rabbits that were immunized with 3 doses of whole-cell NTHi 3655 or KR334. Sera were harvested 4 weeks after the last immunization, and reactivity against recombinant affinity-purified EF-Tu, protein F, and protein D was analyzed by ELISA using HRP-conjugated goat-anti rabbit antibodies. An *E. coli* lysate was included as a negative control. Significance was assessed using the Mann-Whitney *U-*test, and error bars indicate SEM.

We next assessed the NTHi-induced immune response against EF-Tu. Rabbits were immunized with heat-killed whole NTHi bacterial cells 3655 and KR317, followed by enzyme-linked immunosorbent assays (ELISAs) of pre-immune and convalescent anti-NTHi sera against three different NTHi antigens (Figure 2). Recombinant NTHi proteins F and D, both of which are surface-exposed in NTHi and accessible by antibodies, were used as positive controls, whereas an *E. coli* lysate was included as a negative control representing the expression host of recombinant NTHi proteins and possible contaminants from *E. coli* (8, 10). Interestingly, in this particular experimental model, recombinant EF-Tu seemed to be immunodominant resulting in 2-fold more IgG directed against EF-Tu than against proteins F and D.

### The Immunodominant Epitopes of EF-Tu Are Located Within Its Central and C-Terminal Regions

**Figure 3:**
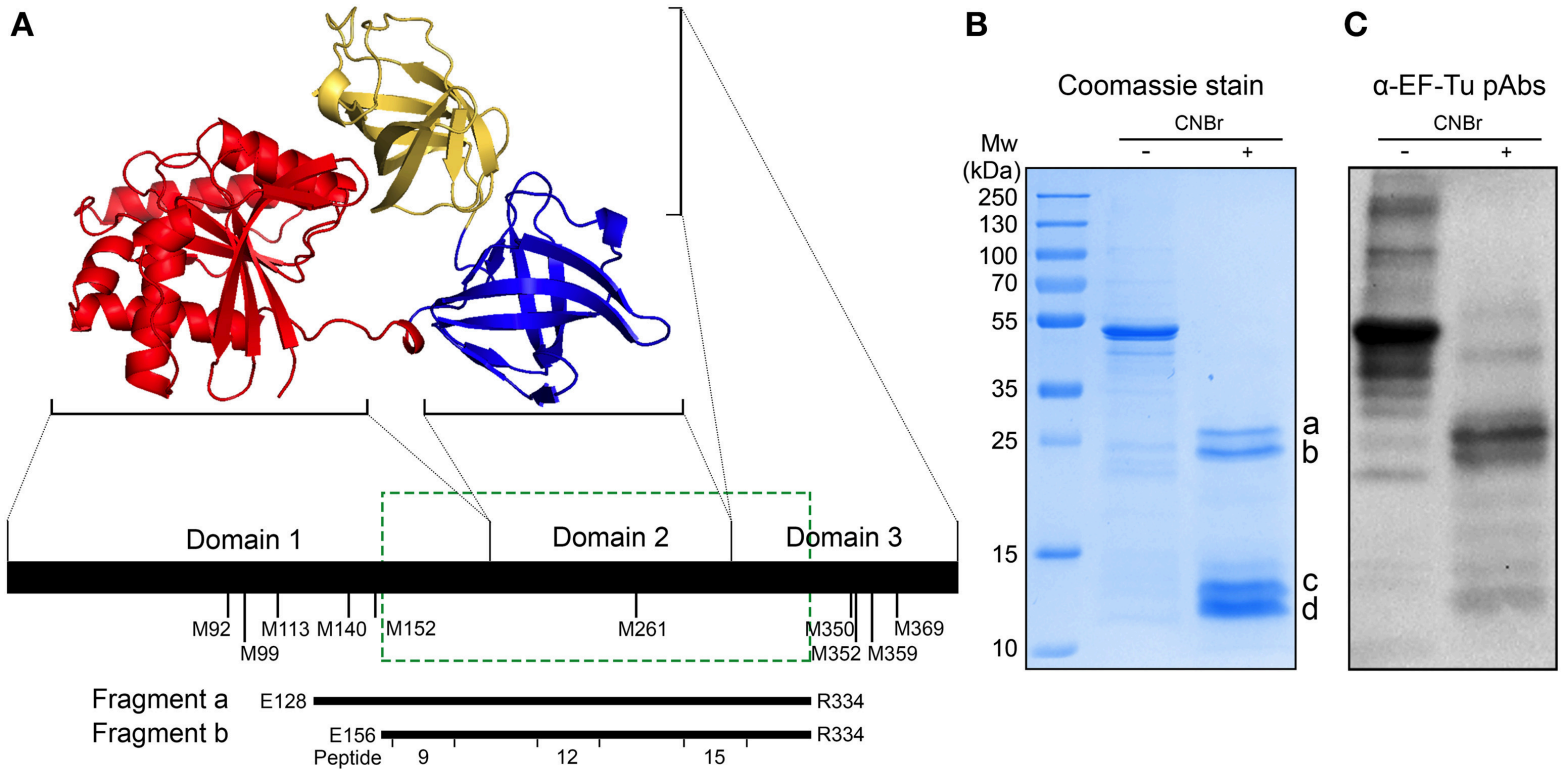
Anti-EF-Tu antibodies mainly target a region comprising the EF-Tu C-terminal part of domain 1, the complete domain 2, and the N-terminal part of domain 3. **(A)** Schematic organization of EF-Tu domains 1–3. The 3-dimensional structure of EF-Tu is based upon PDB 1dg1 (28, 29). Methionine (M) residues where CNBr cleavage can occur are indicated below the horizontal line. Fragments a and b represent two products of EF-Tu cleavage by CNBr, spanning glutamate-128 to arginine-334 (E^128^-R^334^) and E^156^-R^334^ residues of EF-Tu. The green box indicates the experimentally determined immunodominant region of EF-Tu. **(B)** Recombinant NTHi EF-Tu was digested with CNBr and subjected to analysis by SDS-PAGE and Coomassie staining to determine the fragmentation pattern. The indicated cleaved bands a and b were in-gel trypsin digested, excised, and sequenced to identify the corresponding fragments depicted in **(A)**. The CNBr-digested EF-Tu was also subjected to Western blotting with affinity-purified rabbit anti-EF-Tu pAbs **(C)**. Secondary HRP-conjugated anti-IgG pAbs were used for detection in Western blot.

Prokaryotic EF-Tu consists of three domains (12) (Figure 3A). Several segments of EF-Tu are predicted *in silico* to be exposed to the environment and to be antigenic, with the potential to be targeted by host antibodies (Supplementary Figure 1). To identify the immunodominant epitopes of *H. influenzae* EF-Tu, we subjected recombinant NTHi EF-Tu purified from *E. coli* to chemical cleavage by CNBr. This procedure resulted in production of 4 major fragments with molecular weights of 12, 13, 20, and 25 kDa (designated a to d in Figure 3B). Cleaved EF-Tu was subjected to immunoblotting with rabbit anti-EF-Tu pAbs (Figure 3C). Anti-EF-Tu IgG recognized full-length recombinant EF-Tu (≈45.8 KDa) and the two larger fragments. The 25- and 20-kDa EF-Tu fragments (a and b) were subsequently isolated from the gel (Figure 3B) for peptide fingerprinting and identification. These two fragments were identified as spanning NTHi EF-Tu residues glutamate-128 to arginine-334 (E^128^-R^334^) and E^156^-R^334^, respectively (Figure 3A, lower panel). We hence concluded that rabbit serum recognized the middle portion of EF-Tu, comprising the C-terminal part of domain 1, the complete domain 2, and the N-terminal portion of domain 3.

**Figure 4:**
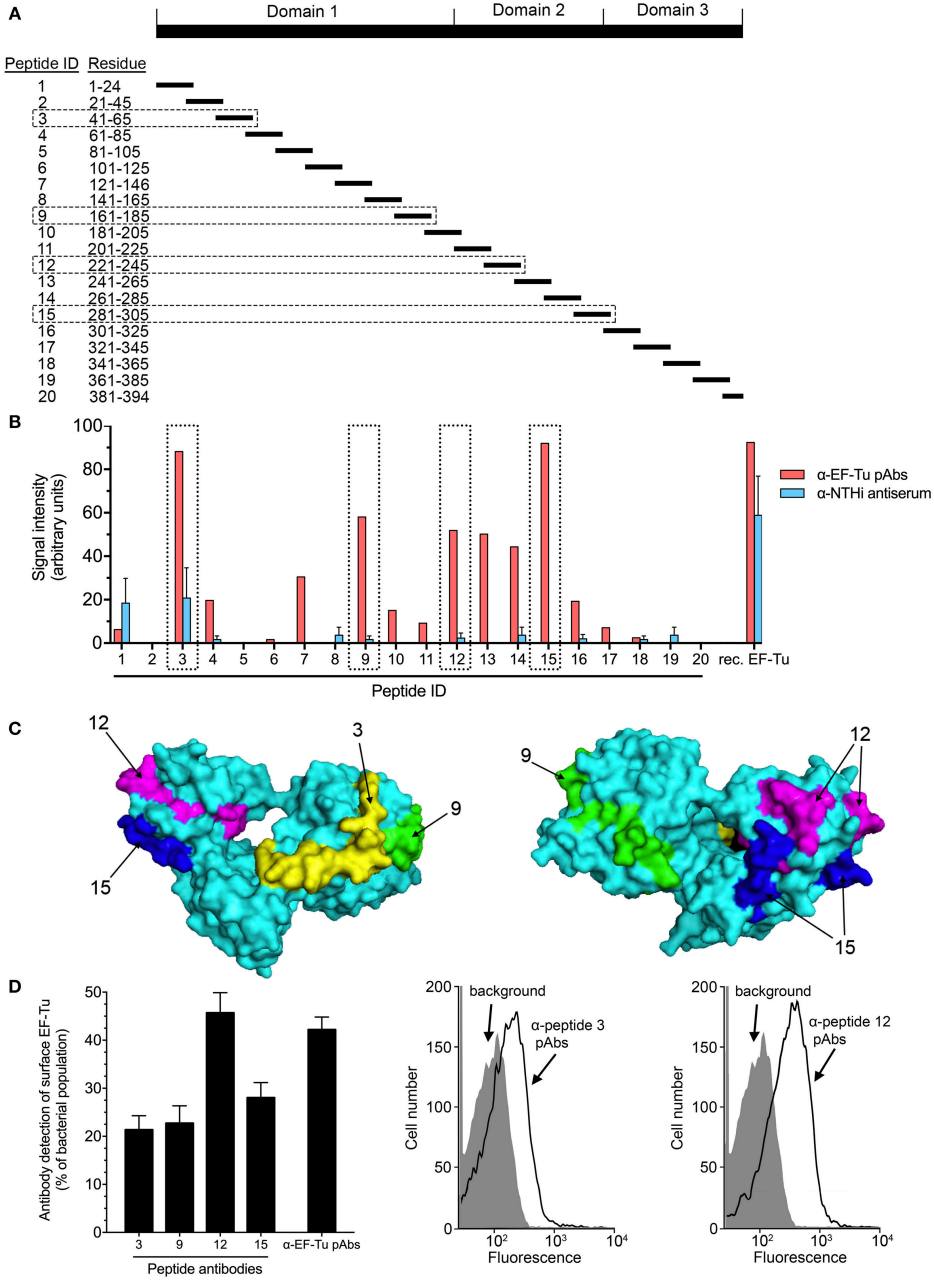
Four main immunoreactive epitopes are detected at the surface of EF-Tu. **(A)** Map of the synthetic peptides covering the EF-Tu protein sequence that were used for epitope mapping. **(B)** The reactivity of anti-EF-Tu pAbs from rabbits (*n* = 4) immunized with recombinant EF-Tu, and of sera from rabbits immunized with whole NTHi (3655 or 334) (*n* = 5) were tested against synthetic EF-Tu peptides using a dot blot (Supplementary Figure 2). Mean values obtained by scanning densitometry are shown. **(C)** The surface-exposed peptides ID 3, 9, 12, and 15 are indicated on the model of the EF-Tu crystal structure (Protein Data Bank entry 1dg1). These peptides corresponded to EF-Tu^41−65^, EF-Tu^161−185^, EF-Tu^221−245^, and EF-Tu^281−305^. Peptides ID 9, 12, and 15 were located within the CNBr-generated fragments indicated in Figure 4A. **(D)** Specific antibodies against peptides ID 3, 9, 12, and 15 recognize EF-Tu on the surface of NTHi 3655, as revealed by flow cytometry. Mean values from 9 experiments are shown. The anti-peptide antibodies were affinity-purified from sera obtained from rabbits immunized with full-length recombinant EF-Tu. Error bars indicate SEM. Representative flow cytometry results are shown for anti-peptide Abs directed against peptide ID 3 and 12. Flow cytometry analysis was performed using NTHi 3655 cells incubated with the anti-EF-Tu IgG or peptide-specific antibodies followed by secondary FITC-conjugated pAbs. Background represents bacteria incubated with secondary antibody only.

To further pin-point the target sequences of anti-EF-Tu IgG, a series of synthetic peptides spanning the entire EF-Tu molecule were synthesized (Figure 4A). Peptide epitope mapping (Supplementary Figure 2) was performed using purified pAbs from rabbits immunized with recombinant EF-Tu or sera from rabbits immunized with whole NTHi. Semi-quantitative dot blot analysis of anti-EF-Tu pAbs revealed that some of the highest levels of reactivity were against peptides ID 3, 9, 12, and 15 (Figure 4B; red bars and boxes), corresponding to sequences EF-Tu^41−65^, EF-Tu^161−185^, EF-Tu^221−245^, and EF-Tu^281−305^, respectively (Figure 4A and Supplementary Figure 3). In contrast, serum from a rabbit immunized with whole NTHi (Figure 2) mainly detected full-length (native) EF-Tu (Figure 4B; blue bars).

Based on the results above, we delineated the potential surface-exposed, antigenic parts of EF-Tu as indicated in Figure 4C. To examine whether the regions corresponding to sequences in peptide ID 3, 9, 12, and 15 were accessible within the EF-Tu molecule on the bacterial surface, we analyzed NTHi by flow cytometry using affinity-purified anti-peptide Abs. All four immunogenic regions were readily detected by the peptide-specific Abs (Figure 4D). Antibodies directed against peptide ID 12 resulted in the strongest signal that was comparable to the one obtained with IgG against full-length recombinant EF-Tu. Taken together, these data revelaed that the immunodominant epitopes of surface-associated EF-Tu are accessible by the host humoral immune system.

### Anti-NTHi EF-Tu Antibodies Are Bactericidal

**Figure 5:**
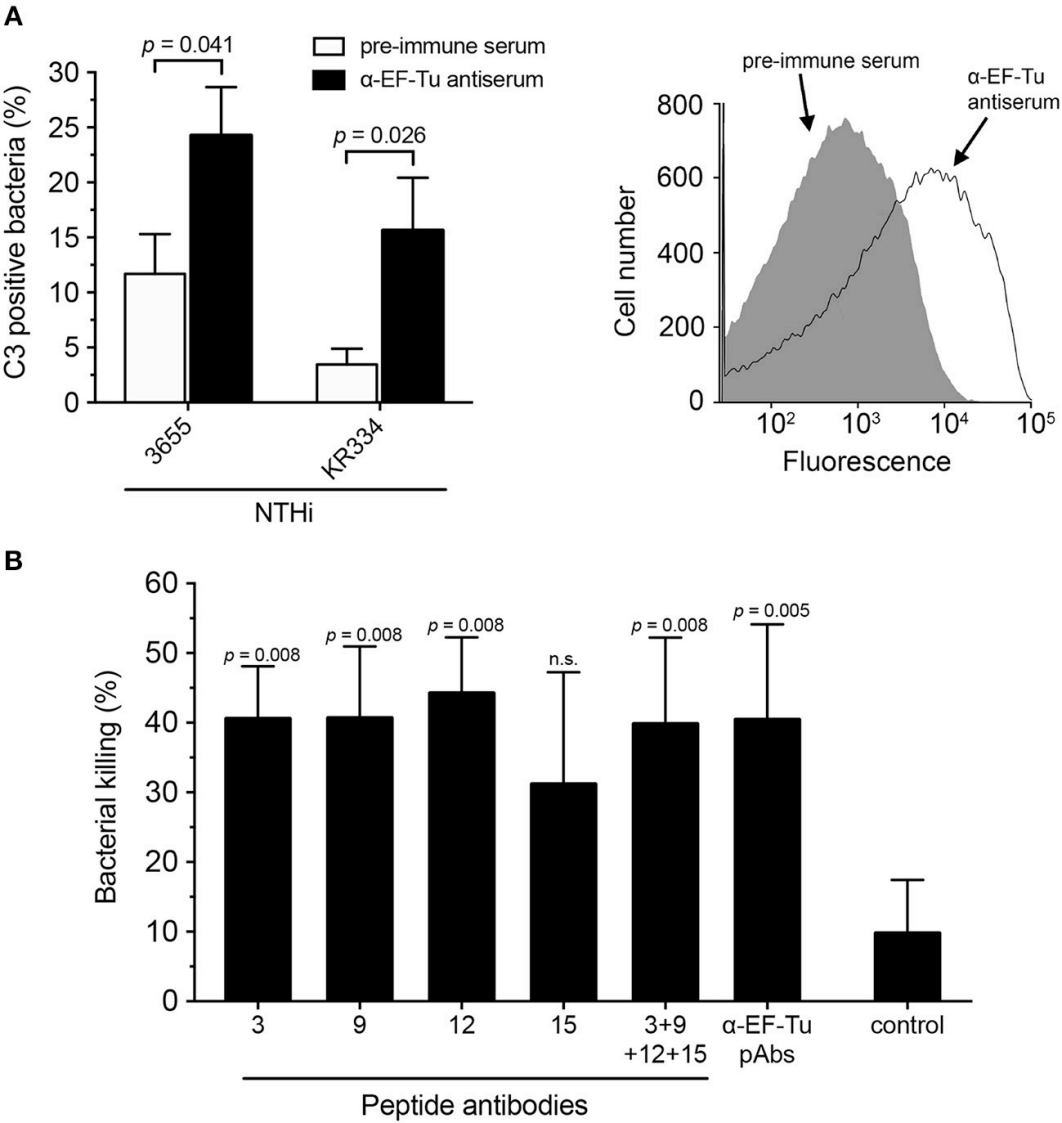
Antibodies directed against EF-Tu are functional and induce bactericidal activity via complement activation. **(A)** C3 deposition was assessed in the presence of rabbit antiserum against full-length EF-Tu. NTHi strains KR334 and 3655 were incubated with EF-Tu antiserum, and baby rabbit complement was used as a complement source. Flow cytometry analysis was performed using a specific anti-C3 antibody. Pre-immune serum was used as a negative control. Mean values from 6 experiments are shown. A representative flow cytometry profile is also shown. Background represents bacteria incubated with the secondary antibody only. **(B)** Serum bactericidal activity (SBA) was performed to assess NTHi killing induced by anti-EF-Tu antibodies. Rabbit antibodies directed against full-length EF-Tu or peptide ID 3, 9, and 12 significantly induced the classical pathway of complement activation in the presence of baby rabbit complement. Bacterial killing represents differences between incubation with active or heat-inactivated baby rabbit complement. Control represents bacterial killing from baby rabbit complement with the non-related anti-OprG pAb detecting a *Pseudomonas aeruginosa* outer membrane protein. Mean values from 5 separate experiments are indicated. Statistical significances were calculated using the Mann-Whitney test. Error bars indicate SEM.

The immune response elicited by immunization with recombinant NTHi EF-Tu prompted us to test whether antibodies directed against EF-Tu can induce complement-dependent killing of NTHi cells. Following the pre-incubation of bacteria with serum from rabbits immunized with recombinant EF-Tu and the addition of an external complement source, C3 deposition was analyzed by flow cytometry (Figure 5A). Clear shifts were observed with EF-Tu antiserum compared to control pre-immune serum samples, suggesting initiation of the classical complement activation pathway.

To validate the findings on C3 deposition, we also performed serum bactericidal activity (SBA) assays. Affinity-purified Abs directed against full-length EF-Tu and peptides ID 3, 9, 12, and 15 were incubated with NTHi followed by the addition of complement. Approximately 40% of NTHi were killed following incubation with antibodies directed against specific surface-exposed parts of EF-Tu (Figure 5B). The serum bactericidal activity of the anti-peptide Abs, except for peptide ID 15, was similar to that of IgG pAbs directed against the full-length EF-Tu molecule. The combination of the four anti-peptide antibodies did not, however, exhibit enhanced efficacy relative to the individual antibodies. Taken together, these data demonstrated that Abs directed against EF-Tu trigger the innate immune defense resulting in complement activation, as evidenced by C3b generation and bacterial killing.

### Anti-NTHi EF-Tu pAbs Recognize Unencapsulated Respiratory Tract Bacteria and Promote Antibody-Dependent Killing

**Figure 6:**
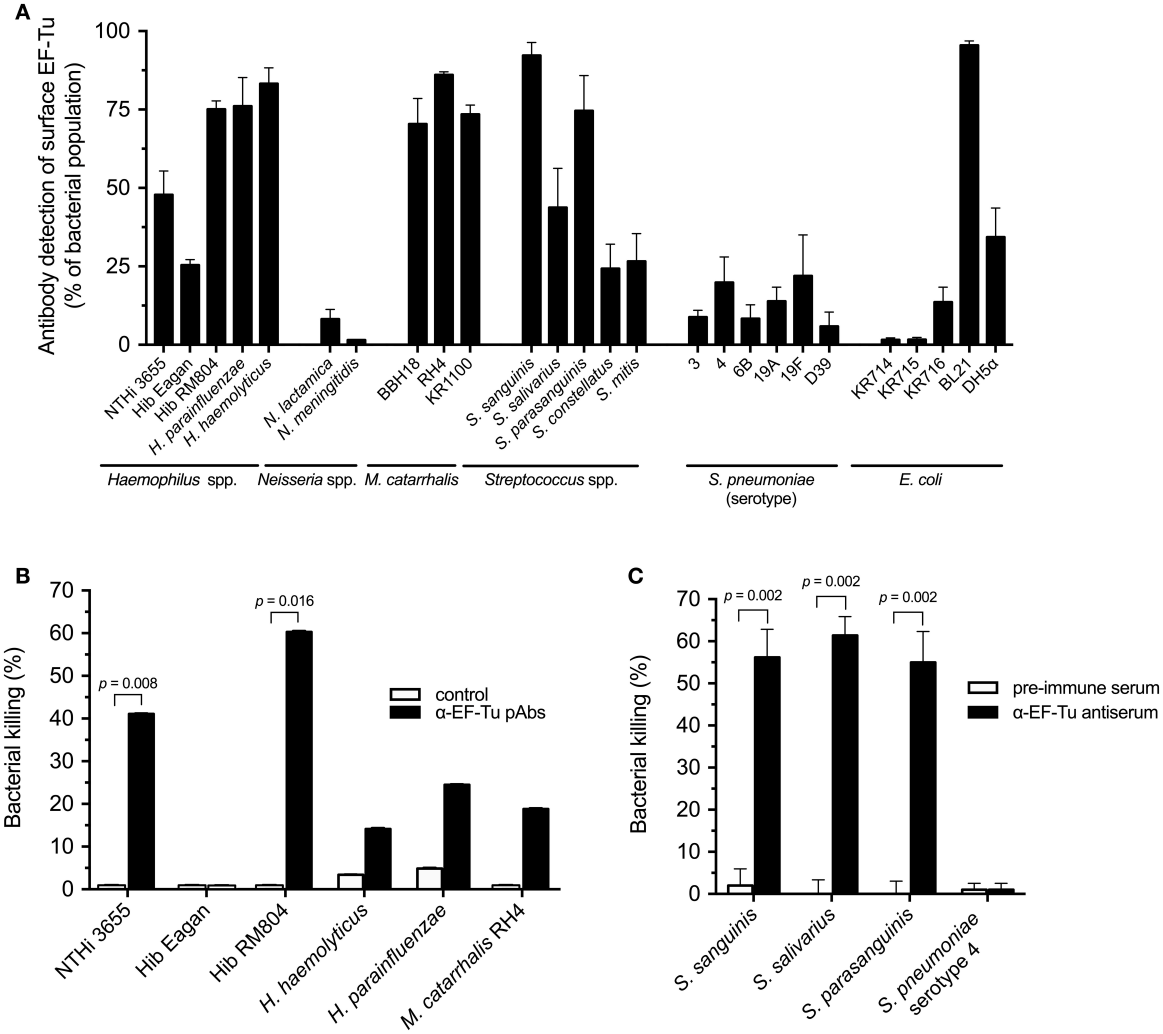
Multiple bacterial species are recognized by NTHi anti-EF-Tu antibodies, but exhibit differential susceptibilities to antibody-mediated bactericidal activity. **(A)** The indicated bacterial species were tested for detection by anti-NTHi EF-Tu pAbs using flow cytometry. Mean values from at least three different experiments are shown. **(B)** Anti-EF-Tu IgG-dependent bactericidal activity against indicated Gram-negative species was tested using the SBA assay. For the SBA assay, the indicated bacterial cells were combined with purified anti-EF-Tu IgG and baby rabbit complement, followed by determination of CFU after overnight incubation. Mean values from 5 independent experiments are shown. **(C)** The Gram-positive *S. sanguinis, S. salivarius*, and *S. parasanguinis*, but not *S. pneumoniae* were killed by HL60 cells when pre-incubated with rabbit anti-NTHi EF-Tu antiserum. Gram-positive bacteria with highest levels of surface-associated EF-Tu, as revealed by flow cytometry, were selected for these experiments. Mean values from 6 independent experiments are shown. *S. pneumoniae* reference strain D39 in panel **A** belongs to serotype 2 (Table 1). Statistical significance was calculated using the Mann-Whitney test. Error bars indicate SEM.

Since anti-EF-Tu pAbs clearly recognized NTHi and elicited a bactericidal effect (Figure 1, 5B), we subsequently investigated whether the anti-EF-Tu pAbs could also recognize other bacterial species with homologous EF-Tu proteins (Supplementary Figure 3). Multiple Gram-negative and Gram-positive bacterial species, including pathogens and commensals, were subjected to flow cytometry analyses followed by assessment of SBA or opsonophagocytosis. Anti-EF-Tu pAbs detected EF-Tu molecules on *H. parainfluenzae, H. haemolyticus*, and *Moraxella catarrhalis*, but did not bind encapsulated *N. meningitidis*. Moreover, the anti-EF-Tu pAbs recognized various unencapsulated *Streptococci* from the oral microbiome, but not the encapsulated *S. pneumoniae* (Figure 6A). Finally, a set of clinical *Escherichia coli* isolates (KR714-716) were not detected by anti-EF-Tu IgG that was in bright contrast to the unencapsulated laboratory strains *E. coli* BL21 and DH5α that significantly carried EF-Tu at the surface.

Subsequent SBA assays revealed that antibodies against NTHi EF-Tu promoted the killing of NTHi and unencapsulated Hib RM804 (Figure 6B), but not of the encapsulated Hib Eagan that did not have any surface-exposed EF-Tu. In addition, *H. haemolyticus, H. parainfluenzae*, and *M. catarrhalis* were targeted for antibody-dependent complement attack, but at levels lower than NTHi and RM804 (Figure 6B). The effects of anti-NTHi EF-Tu IgG against Gram-positive, unencapsulated *S. sanguinis, S. salivarius*, and *S. parasanguinis* were also tested in an opsonophagocytosis assay (OPA) using the phagocytic cell line HL60 pre-activated with dimethylformamide. Phagocytes exhibited significant killing activity against the unencapsulated *Streptococcus* species, but not against encapsulated *S. pneumoniae* (Figure 6C). Taken together, IgG directed against NTHi EF-Tu recognized most unencapsulated bacteria derived from the upper respiratory tract microbiome and promoted EF-Tu IgG-dependent killing via SBA and opsonophagocytosis.

**Table 1:** Bacterial species used in the present study.

**Reference strain or clinical isolate**	**Serotype/group**	**Number**	**Site of origin**	**Clinical diagnosis/remarks**	**Age (year)**	**Reference**
**GRAM-NEGATIVE SPECIES**						
*H. influenzae*	Type b (Hib)	Eagan (18095)	Cerebrospinal fluid	Meningitis/ encapuslated		CCUG^a^
	Type b (Hib)	RM804		Capsule-deficient mutant of Eagan		(35)
	Type b (Hib)	MinnA	Cerebrospinal fluid	Laboratory reference strain		(36)
Non-typeable *H. influenzae*		KR317	Nasopharynx	Bronchitis	55	This study
(NTHi)		KR318	Nasopharynx	Bronchitis	49	This study
		KR332	Nasopharynx	Upper respiratory tract infection	3	This study
		KR333	Nasopharynx	Sinusitis	68	This study
		KR334	Nasopharynx	Conjunctivitis	2	This study
		3655	Middle ear	Acute otitis media		(37)
*H. parainfluenzae*		13788	Oral cavity	Reference strain		CCUG
*H. haemolyticus*		KR161	Throat	Normal flora		This study
*Moraxella catarrhalis*		BBH18	Blood	Sepsis		(38)
		RH4	Blood	Sepsis		(39)
		KR1100	Nasopharynx	Bronchitis	85	This study
*Neisseria lactamica*		23972		Reference strain		ATCC^b^
*N. meningitides*	Serogroup A	3269	Spinal fluid	Meningitis		CCUG
	Serogroup B	MC58				National Reference lab., Örebro, Sweden
	Serogroup C	KR822	Spinal fluid	Meningitis		This study
	Serogroup C	FAM20				(40)
*Escherichia coli*		KR714	Urine	Urinary tract infection	6	This study
		KR715	Urine	Urinary tract infection	63	This study
		KR716	Urine	Urinary tract infection	59	This study
		BL21		Laboratory strain	
		DH5α		Laboratory strain	
**GRAM-POSITIVE SPECIES**						
*Streptococcus pneumoniae*	ST2^c^	D39		Reference strain		ATCC
	ST3	6303		Reference strain		ATCC
	ST4	KR440	Blood	Pneumonia, sepsis	73	This study
	ST6B	1350	Cerebrospinal fluid	Meningits, otitis/ Reference strain		CCUG
	ST19A	KR1148	Nasopharynx	AOM	2	This study
	ST19F	49619		Reference strain		ATCC
*S. sanguinis*		KR1143		Normal flora	60	This study
*S. parasanguinis*		KR1144		Normal flora	45	This study
*S. salivarius*		KR1145		Normal flora	68	This study
*S. constellatus*		KR1146		Normal flora	64	This study
*S. mitis*		KR1147		Normal flora	64	This study

a * CCUG; Culture collection University of Gothenburg (Sweden) (www.ccug.se)*.

b *ATCC; American type culture collection (www.atcc.org)*.

c *ST; serotype*.

## Discussion

We found that antibody-accessible EF-Tu is associated with the surface of NTHi cells using flow cytometry and TEM (Figures 1A–C). This is in agreement with previous observations with other bacteria from the gastrointestinal and respiratory tracts displaying surface-associated EF-Tu (15–19). Our findings support the role of EF-Tu as a moonlighting protein that possibly mediates interactions with the host extracellular matrix and components of the innate immunity (21, 24). In conjunction with previous reports, our observations underscore the importance of EF-Tu surface exposure in bacterial fitness and virulence.

Considering the immunogenicity of surface-associated EF-Tu in various mammalian hosts, we sought to identify the immunodominant sequences and surface-exposed parts of NTHi EF-Tu. Epitope mapping of NTHi EF-Tu based on CNBr-fragmented EF-Tu and peptide libraries unveiled several immunogenic regions, corresponding to peptides ID 3, 9, 12, and 15 (Figures 3, 4 and Supplementary Figure 2). The antigenic properties of these peptide sequences were predicted *in silico* by B-cell epitope analysis (Supplementary Figure 1A), and was in concordance with our mapping data. Furthermore, using bioinformatic analyses, these peptides were also predicted to be accessible at the protein surface (Figure 4C and Supplementary Figure 1). Surface accessible antigen determinants or B-cell epitopes are crucial for an appropriate antibody response (30), and these collective findings establish peptides ID 3, 9, 12, and 15 as immunodominant sequences of NTHi EF-Tu. Similarly, Kolberg *et al*. showed that a monoclonal mouse IgG raised against pneumococcal EF-Tu recognized EF-Tu domains 2 and 3 (31). In contrast, Pyclik *et al*. recently studied EF-Tu from *Streptococcus agalactiae* and found two sequences to be recognized by human antibodies; ^28^LTAAITTVLARRLP^41^ (slightly overlapping NTHi EF-Tu peptide ID 3 on domain 1) and ^294^ GQVLAKPGSINPHTKF^309^ (corresponding to NTHi EF-Tu peptide ID 15). This discrepancy could be due to hitherto unknown proteolytic processing events on the bacterial surface (24). Interestingly, we found peptide ID 3 to be the only peptide detected by both α-EF-Tu pAb and the α-NTHi serum (Figure 4B). IgG antibodies directed against peptide ID 3 provided, however, the weakest signal in flow cytometry analysis of whole NTHi (Figure 4D), suggesting that the protein structure is slightly altered *in vivo* with other parts (epitopes) exposed of the EF-Tu molecule.

The immunoreactive properties of EF-Tu has been thoroughly evaluated (24, 32, 33). However, a high titer of specific antibodies does not always translate to increased protection of the host (34). An example of this is the study done by Carrasco *et al*. who showed that EF-Tu in *Borrelia burgdorferi* is highly immunogenic, but the antibodies produced are not bactericidal during Lyme borreliosis (16). This is alluded to the finding that the EF-Tu is not accessible at the *B. burgdorferi* bacterial surface. We found that serum from rabbits immunized with recombinant NTHi EF-Tu, when supplemented with active baby rabbit complement, was able to significantly increase the deposition of complement factor C3 on the bacterial surface (Figure 5A). In addition, purified antibodies against full-length NTHi EF-Tu and peptide ID 3, 9 and 12 significantly increased bacterial killing by complement activation in the SBA assay (Figure 5B). These results can be interpreted as further evidence of that EF-Tu and the indicated peptide sequences are indeed accessible at the bacterial surface. This also makes it interesting to further study whether immunization with NTHi EF-Tu can generate protection against colonization or infection with NTHi *in vivo*.

The high similarity (and function) of EF-Tu between different bacterial species (Supplementary Figure 3) warrants the speculation of cross-reactivity, especially between *Haemophilus* species that share EF-Tu sequence, and its potential implications on the microbiome. Flow cytometry analysis indicated that antibodies against NTHi EF-Tu also cross-react with various unencapsulated species, supporting surface display of EF-Tu in general. As expected, EF-Tu was not detectable on the surface of most encapsulated bacteria, including *H. influenzae* type b (Hib) *N. meningitides*, or pneumococci (Figures 1D–F, **6A**). This underscores previous suggestions that the capsule of pneumocci and meningococci shields surface-associated EF-Tu from antibody dectection (31). There was a clear correlation between EF-Tu recognition and differences in antibody-dependent bacterial killing (Figures 6A,B) for *H. influenzae* strains. However, despite being recognized by anti-EF-Tu antibodies at the same level as NTHi, *H. haemolyticus, H. parainfluenzae*, and *M. catarrhalis* were less susceptible to the antibody-dependent bactericidal activity as compared to NTHi suggesting that C1q binding and further activation of the classical pathway of complement activation was not maximally initiated. Our results thus suggested that species-specific differences exist regarding EF-Tu surface exposure. However, the full extent of cross-reacivity with the upper respiratory tract microbiota, but also the gut microbiome must be further studied. In particular, commensal bacteria not expressing IgA1 protease might be subjected to a negative selective pressure by secreted IgA specific for surface-exposed EF-Tu.

In conclusion, we have shown that EF-Tu moonlights at the surface of NTHi, and that the protein is highly immunogenic with immunodominant epitopes residing primarily at the C-terminal half of the molecule. IgG raised against NTHi EF-Tu can cross-react with and promote antibody-dependent killing of other bacterial species, albeit at rates different from those observed for NTHi. We propose that the adaptive immune response against surface EF-Tu is highly important in the context of respiratory tract infections in order to protect the host from attack and consequently infection.

## Materials and Methods

### Bacteria and Culture Conditions

Bacteria used in the present study are listed in Table 1. NTHi was grown at 37°C in a humid atmosphere containing 5% CO_2_ on chocolate agar or in brain heart infusion (BHI) broth supplemented with 2 μg/mL nicotinamide adenine dinucleotide NAD (Sigma-Aldrich, St Louis, MO, United States) and 10 μg/mL hemin (Merck, Darmstadt, Germany). *E. coli* was cultured in Luria-Bertani (LB) broth or on LB agar. All other strains were cultured on chocolate agar or in BHI at 37°C with 5% CO_2_. Clinical isolates were obtained from Clinical Microbiology (Laboratory Medicine, Lund, Sweden). Type strains were from the American Type Culture Collection (ATCC; Manassas, VA, United States) or Culture Collection of the University of Gothenburg (CCUG; Department of Clinical Bacteriology, Sahlgrenska Hospital, Gothenburg, Sweden).

### Production of Recombinant NTHi EF-Tu and Synthesis of EF-Tu Peptides

The open reading frame of the gene encoding full-length NTHi EF-Tu (EDJ92442.1) was amplified from NTHi 3655 genomic DNA using the primer pair 5′-GGGGCGGATCCGATGTCTAAAGAAAAATTTGAACGTA-3′/5′-GGCGGAAGCTTTTTGATGATTTTCGCAACAACGCCA-3′ containing restriction enzyme sites *Bam*HI and *Hin*dIII (underlined), respectively. Following restriction enzyme digestion, the resulting DNA fragment (1214 base pairs) was cloned into the expression vector pET26(b)+ (Novagen, Merck Darmstadt, Germany) for recombinant protein production as described previously (8). Briefly, the resulting plasmid was transformed into *E. coli* DH5α, followed by DNA sequencing. Recombinant proteins were thereafter produced in *E. coli* BL21 (DE3) and purified by affinity chromatography using Ni-NTA agarose. For NTHi epitope mapping, 20- to 25-residue-long peptides, overlapping by 5 residues and together covering the entire EF-Tu sequence, were synthesized by Genscript (Piscataway, NJ). These peptides were also used for affinity-purification of Abs from the rabbit anti-EF-Tu antiserum as described below.

### Antisera and Antibody Preparation

Rabbit anti-NTHi 3655, anti-NTHi 334, anti-EF-Tu sera and rabbit anti-EF-Tu pAbs were prepared as previously described (8) with some modifications. Briefly, rabbits were immunized subcutaneously with 200 μg of recombinant EF-Tu in 0.5 ml saline or with 10^9^ CFU of indicated heat-killed NTHi strains with 0.5 ml incomplete Freund's adjuvant. The animals were boosted 3 times every 4 weeks with alum used as an adjuvant. Blood was drawn 2 weeks after the last immunization. Rabbit pAbs against EF-Tu and antibodies against specific EF-Tu peptides (Supplementary Figure 3A) were further affinity purified using EF-Tu or synthetic peptides (peptide ID: 3, 9, 12, and 15) coupled to CNBr-activated Sepharose^TM^ (GE Healthcare Biosciences, Chicago, IL).

### Flow Cytometry

Bacteria grown for 3 h or overnight at 35.5°C with 5% CO_2_, were adjusted to 10^9^ CFU/ml in PBS containing 1% bovine serum albumin (BSA). Samples containing 2 × 10^7^ bacteria were incubated for 1 h on ice with 2 μg of rabbit anti-EF-Tu pAb or EF-Tu peptide specific antibodies, washed, and incubated for 20 min on ice with fluorescein isothiocyanate (FITC)-conjugated swine anti-rabbit antibodies (Dako, Glostrup, Denmark). Samples were washed with phosphate-buffered saline (PBS) and resuspended in 300 μl PBS for analysis on a FACSverse^TM^ flow cytometer (Becton-Dickson, Franklin Lakes, NJ).

### Transmission Electron Microscopy (TEM)

TEM was used to visualize the localization of EF-Tu on the bacterial surface. The *H. influenzae* NTHi 3655, NTHi KR334, Hib Eagan, and Hib RM804 strains were incubated with gold-labeled rabbit anti-EF-Tu pAbs, subjected to negative staining with uranyl formate, and visualized using a Jeol JEM 1230 electron microscope (JEOL, Tokyo, Japan) operated at 60 kV accelerating voltage. Images were recorded with a Gatan Multiscan 791 CCD camera (Gatan, Pleasanton, CA).

### ELISA

Following the addition of 0.5 μg of purified recombinant EF-Tu, protein F (8), or protein D (41) in 0.1 M Tris (pH 9) per well, Nunc PolySorp 96-well microtiter plates (Thermo Fisher Scientific, Waltham, MA) were incubated overnight at 4°C. After 4 washes, the plates were blocked for 1 h at RT with 250 μl PBS with tween 20 (PBST) with 1% BSA per well. After washing, the samples were incubated for 1 h at RT with sera diluted 1:100 in PBST with 2.5% BSA and subsequently washed 3 times. HRP-conjugated goat anti-rabbit pAbs (Dako, Glostrup, Denmark) were added for 1 h at RT, with 4 washes following the incubation. At all steps, each wash was performed for 5 min using PBST. Antibody-antigen complexes were detected using a hydrogen peroxide/3,3′,5,5′-tetramethylbenzidine (TMB) substrate solution, with reactions stopped with 1 M sulfuric acid, followed by determination of the absorbance at optical density 450 nm.

### Cyanogen Bromide Digestion

Full-length EF-Tu was treated with 1 μg cyanogen bromide (CNBr) in 70% formic acid per 5 μg of protein. Samples were incubated for 2 h at 25°C in a vacuum centrifuge. Lyophilized samples were resuspended in phosphate-buffered saline (PBS).

### SDS-PAGE and Western Blotting

Samples with full-length EF-Tu or CNBr-digested EF-Tu were separated on a 12% polyacrylamide gel and either stained with Coomassie Brilliant Blue R-250 (Bio-Rad, Munich, Germany) or transferred onto a 0.45-μm Immobilon-P PVDF Membrane (Millipore, Bedford, MA, United States) at 16 V for 15 h. Following blocking in 5% skim milk in PBST, membranes were incubated at room temperature (RT) for 1 h with rabbit α-EF-Tu pAbs diluted 1:1,000 in 5 ml PBST with 5% skim milk. Following three washes in PBS, the membranes were incubated for 1 h with horseradish peroxidase (HRP)-conjugated swine anti-rabbit pAbs (Dako, Glostrup, Denmark). The membranes were thereafter washed in PBS with 0.05% Tween 20 (PBST), developed using Pierce^TM^ Enhanced Chemiluminescence (ECL) Western Blotting Substrate (Thermo Scientific, Waltham, MA), and visualized on a BioRad ChemiDoc^TM^.

### Peptide-Based Epitope Mapping

To identify epitopes recognized by the anti-EF-Tu IgG, EF-Tu peptides (20 μg; peptide ID 1-20; Figure 4A) or full-length EF-Tu (0.05, 0.5 and 5 μg) were coated onto nitrocellulose (NC) membranes. The membranes were dried for 30 min at 37°C, stained with Ponceuau S, and thereafter blocked for 1 h at RT in PBST with 1% BSA and 1% casein. The membranes were then incubated overnight at 4°C with sera diluted 1:100 in PBST with 1% BSA and 1% casein. The membranes were incubated with HRP-conjugated swine anti-rabbit pAbs (Dako) for 20 min at RT, with 4 washes (10 min each) in PBST performed prior to and following the incubation. Membranes were developed using Pierce^TM^ ECL Western Blot substrate and visualized on a BioRad ChemiDoc^TM^. Pixel densities of the dot blot images were assessed using ImageJ® version 1.51.

### Structural Modeling

The 3D structure of NTHi EF-Tu was modeled by SWISS-MODEL (42–45) automated server against homologous templates available in the Protein Data Bank (PDB; available at: www.pymol.org/ http:/www.rcsb.org). Three-dimensional model were elucidated using the program PyMOL (available at: http://www.pymol.org/).

### Serum Bactericidal Activity (SBA) and Determination of C3 Deposition

Susceptibility of antibody-exposed bacterial cells to complement-mediated killing was measured using a modified SBA assay as previously described (8). Briefly, 4 × 10^4^ CFU of indicated bacterial strains were resuspended in Hank's balanced salt solution with 2% heat-inactivated baby rabbit complement (Nordic BioSite AB, Täby, Sweden) followed by incubation with 2.5 μg rabbit anti-EF-Tu pAbs or peptide-specific antibodies for 30 min at RT. In some experiments, anti-OprG pAb detecting a *Pseudomonas aeruginosa* outer membrane protein (Riesbeck *et al*., unpublished) was included as a control pAb not recognizing NTHi. After the addition of active or heat-inactivated baby rabbit complement to a final concentration of 2.5%, samples were incubated for 1 h at 37°C with gentle shaking. Aliquots (10 μl) from the reaction mixtures were plated on chocolate agar, and CFUs were determined after overnight incubation. To determine C3 deposition at the bacterial surface, NTHi 3655 cells were incubated with pre-immune or anti-EF-Tu sera from the same rabbit, followed by the addition of 4% baby rabbit complement as described above. The cells were thereafter stained with FITC-conjugated goat anti-rabbit C3 pAbs (MP Biomedicals, Santa Ana, CA, United States), followed by analysis using a FACSverse^TM^ flow cytometer.

### Opsonophagocytosis Assay (OPA)

Antibody functionality and bacterial survival in opsonophagocytosis was determined by an OPA as previously described (46). HL60 cells (kindly provided by Prof. Urban Gullberg, Lund University) were cultured in RPMI 1640 medium supplemented with 10% fetal bovine serum and GlutaMAX (Gibco, Life Technologies, Carlsbad, CA). Cells were differentiated by adding 0.91% dimethylformamide for 5–6 days. Granulocytes expressing CD35 were detected by flow cytometry and viability was determined using trypan blue staining. Bacteria were added in duplicates to 3-fold serial serum dilutions, starting at 1:4 serum concentration, and incubated in microtiter plates for 30 min at 700 rpm to promote antibody binding. Following the addition of differentiated HL60 cells and baby rabbit complement serum, the plates were incubated for 45 min at 37°C with 5% CO_2_ and at 700 rpm to allow for phagocytosis. The contents were thereafter transferred to blood agar plates and grown overnight. The assays were done using EF-Tu antiserum and pre-immune serum as the negative control. Killing by effector cells was verified in each experiment using pneumococci and in-house quality control serum from volunteers immunized with a pneumococcal conjugate vaccine (Prevenar13).

### Statistics

Mann-Whitney U-test was used for nonparametric data sets and differences were considered statistically significant at p ≤ 0.05. All analyses were performed using GraphPad PrismR version 7.0 (GraphPad Software, La Jolla, CA).

### Ethics Statement

Ethical permit M106-16 (date 2016-09-28) was obtained for immunization of rabbits (Malmö/ Lund Tingsrätt, Sweden). The use of human sera (controls in the OPA) was approved by the Regional Ethics Board at Lund University Hospital (2012/86). Informed consent was obtained from participants.

## Author Contributions

Y-CS and KR planned the study. OT, Y-CS, MB, NL, BD, VA, and FJ contributed to the experimental work. OT, Y-CS, and KR wrote the manuscript.

### Conflict of Interest Statement

The authors declare that the research was conducted in the absence of any commercial or financial relationships that could be construed as a potential conflict of interest.
