# Supplementary material for: EF-Tu From Non-typeable Haemophilus influenzae Is an Immunogenic Surface-Exposed Protein Targeted by Bactericidal Antibodies
Source: Front Immunol. 2018 Dec 18;9:2910. doi: 10.3389/fimmu.2018.02910 (PMC6305414; doi:10.3389/fimmu.2018.02910)
Supplement: Supplementary file 1 [file Data_Sheet_1.docx]

*Supplementary Material

Elongation-factor Tu from non-typeable Haemophilus influenzae is an immunogenic surface-exposed protein targeted by bactericidal antibodies

**Oskar Thofte, Yu Ching Su, Marta Brant, Nils Littorin, Benjamin Luke Duell, Vera Alvarado, Farshid Jalalvand, and Kristian Riesbeck^*^**

*** Correspondence:** Corresponding Author: kristian.riesbeck@med.lu.se

# Supplementary Figures and Tables

**1.1 Supplementary Figures**

**
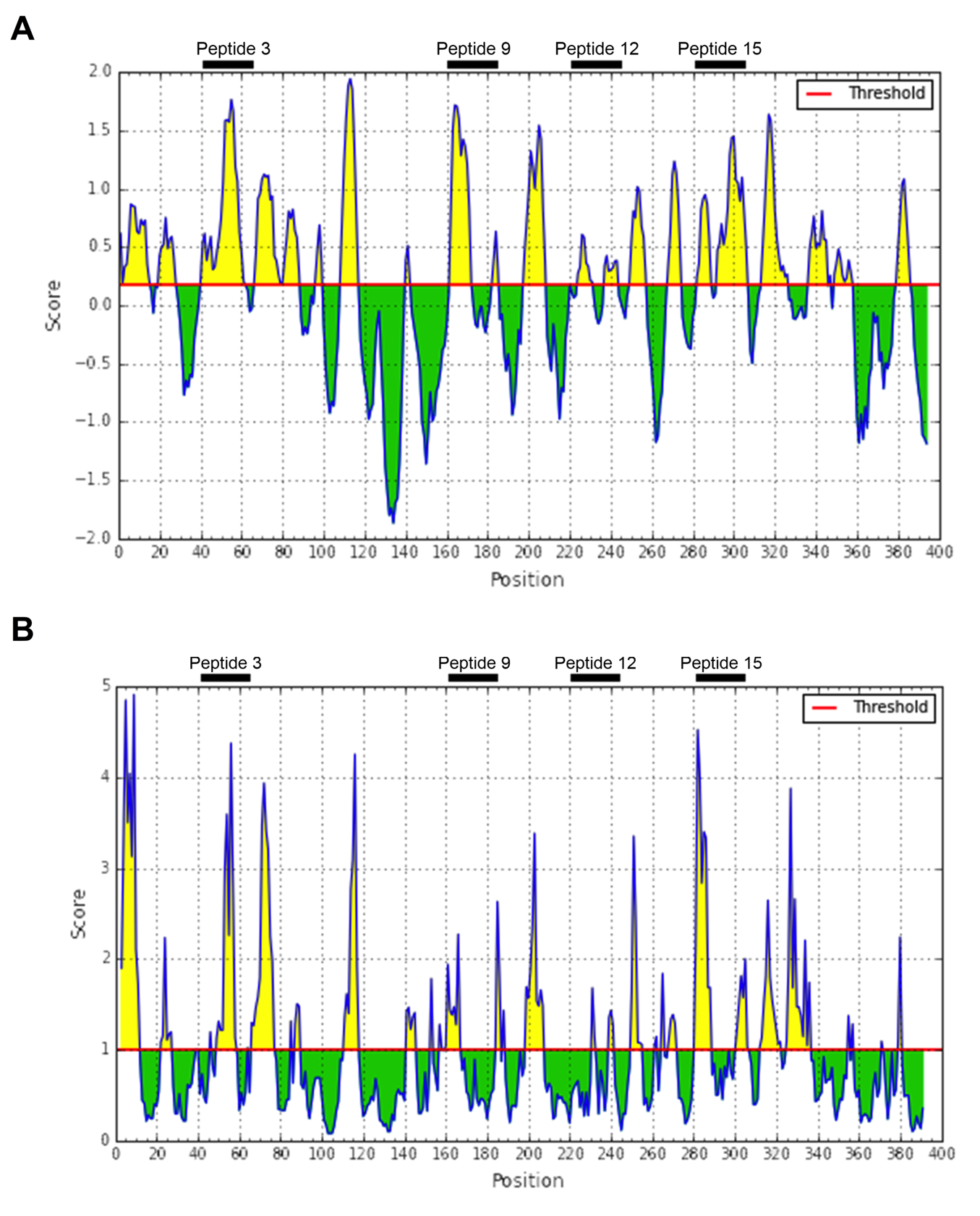
A**

**B**

**Supplementary Figure 1.** *In silico* prediction of antibody epitopes of full-length EF-Tu. (A) The distribution of B-cell linear epitopes on NTHi EF-Tu was predicted using Bepipred Linear Epitope Prediction (1), with a window size of 7 and a threshold value of 0.172. (B) Analysis of surface accessibility was performed using the Emini surface accessibility tool, with a window size of 3 and a threshold value of 1.0 (2).

**
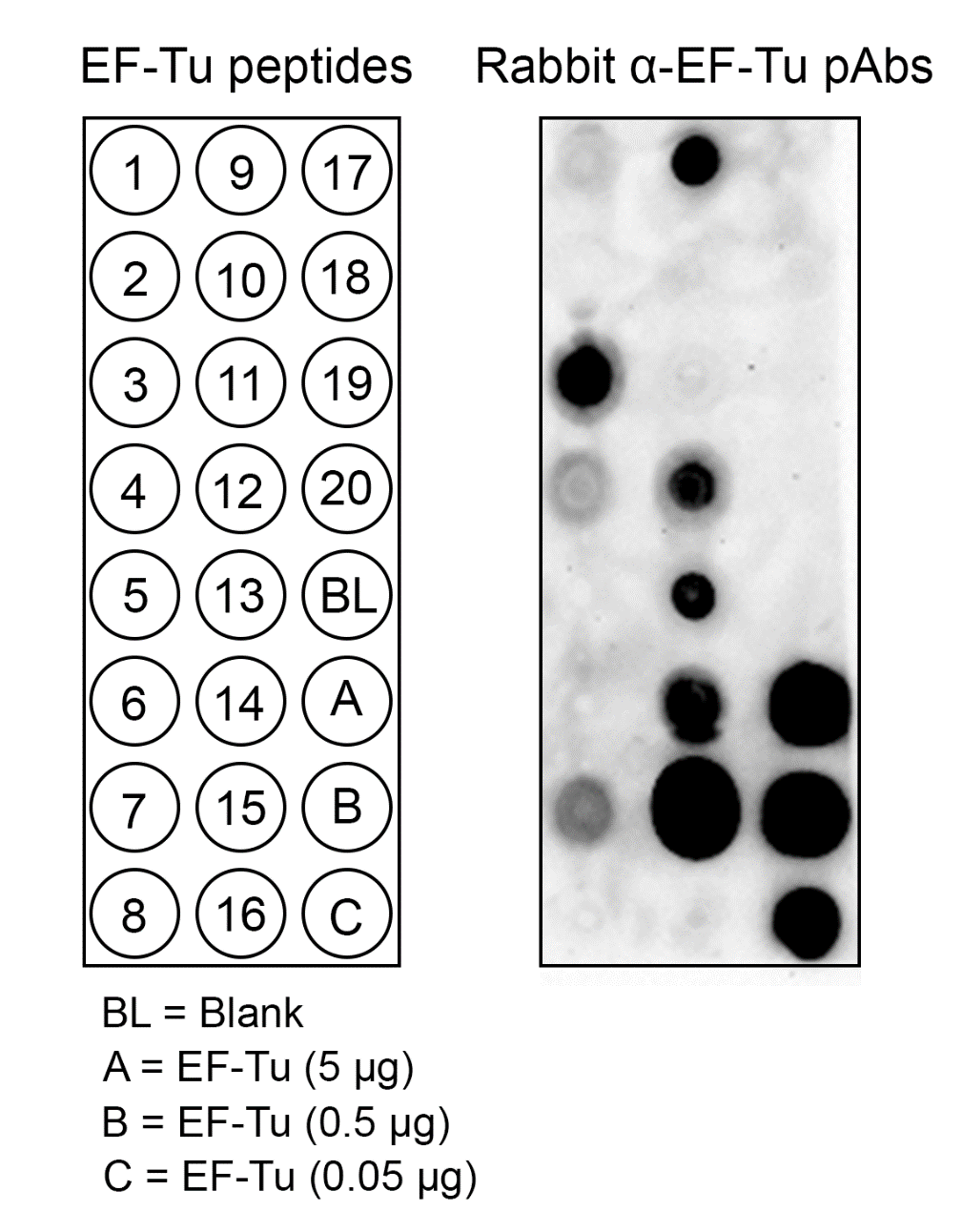
**

**Supplementary Figure 2.** An example of peptide mapping of EF-Tu immunodominant epitopes recognized by rabbit antibodies directed against EF-Tu. Epitope mapping was performed using dot blots of EF-Tu peptides probed with affinity-purified pAbs from rabbits immunized with recombinant NTHi EF-Tu produced in *E. coli*. Anti-EF-Tu pAbs were incubated with filters containing spotted peptides, followed by incubation with HRP-conjugated anti-rabbit IgG antibodies. Semi quantitative analysis of detected spot signal densities was performed and is shown in **Figure 4B**.

**A**


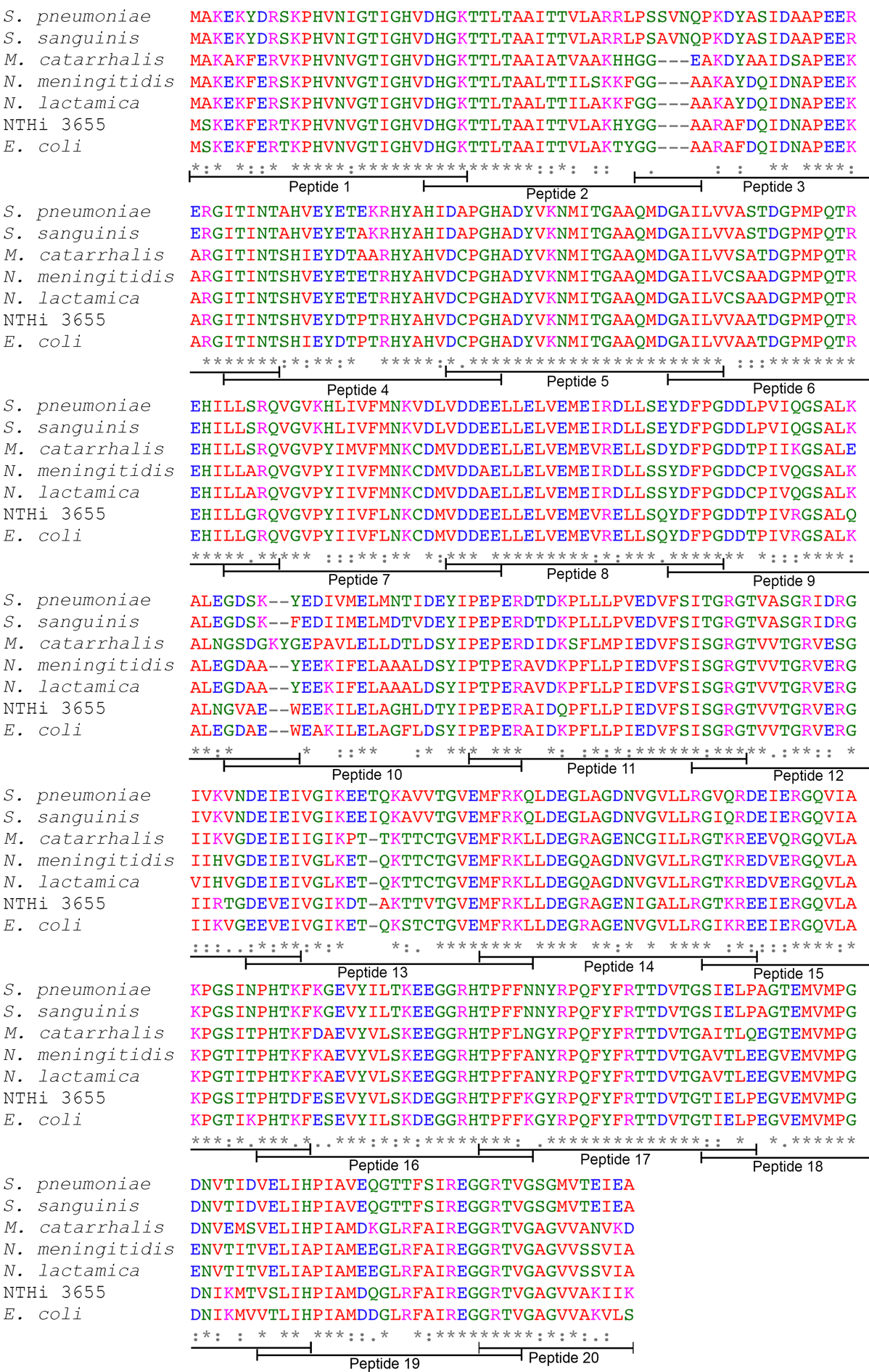


**B**


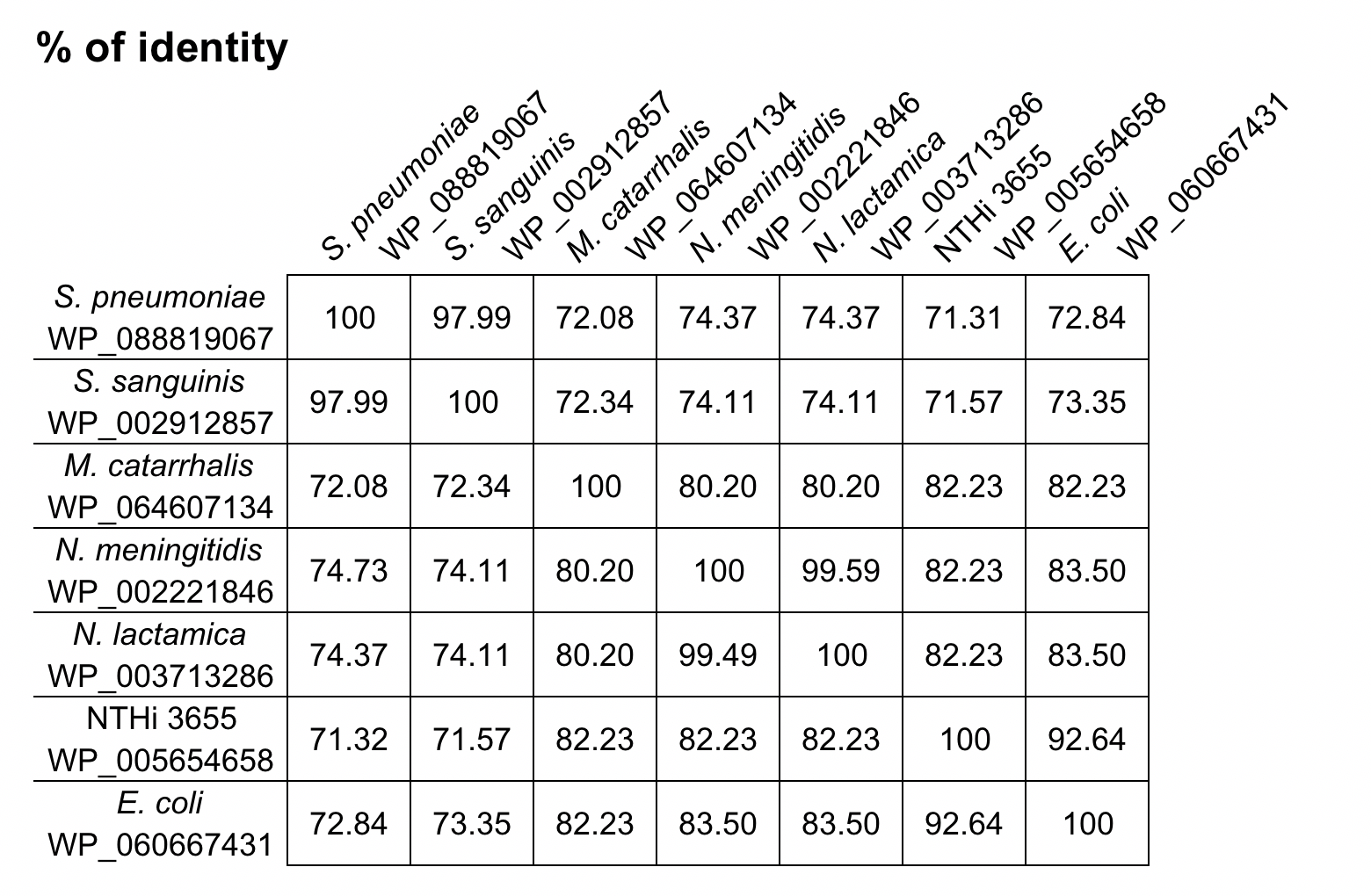


**Supplementary Figure 3.** Alignment and similarity of EF-Tu from selected Gram-negative and Gram-positive bacterial species. (A) Alignment of amino acid sequences of EF-Tu from *S.* *pneumoniae*, *S. sanguinis*, *M. catarrhalis*, *N. meningitides*, *N. lactamica*, NTHi 3655 and *E. coli*. Sequences of EF-Tu peptides used in the study are marked with braces. (B) Analysis of EF-Tu sequence similarities. The alignment and analysis were performed using Clustal Omega (available at: https://www.ebi.ac.uk/).

**Supplementary references**

1. Larsen JEP, Lund O, Nielsen M. Improved method for predicting linear B-cell epitopes. *Immunome Res* (2006) **2**:2. doi:10.1186/1745-7580-2-2

2. Emini EA, Hughes JV, Perlow DS, Boger J. Induction of hepatitis A virus-neutralizing antibody by a virus-specific synthetic peptide. *J Virol* (1985) **55**:836–839.
